# Aerobic Methanotrophy and Co-occurrence Networks of a Tropical Rainforest and Oil Palm Plantations in Malaysia

**DOI:** 10.1007/s00248-021-01908-3

**Published:** 2021-10-30

**Authors:** Adrian Ho, Ali Tan Kee Zuan, Lucas W. Mendes, Hyo Jung Lee, Zufarzaana Zulkeflee, Hester van Dijk, Pil Joo Kim, Marcus A. Horn

**Affiliations:** 1 Institute for Microbiology, Leibniz Universität Hannover, Hannover, Germany; 2 Department of Land Management, Faculty of Agriculture, Universiti Putra Malaysia, Seri Kembangan, Selangor Malaysia; 3 Center for Nuclear Energy in Agriculture, University of São Paulo (CENA-USP), Sao Paulo, Brazil; 4 Department of Biology, Kunsan National University, Gunsan, South Korea; 5 Department of Environment, Faculty of Forestry and Environment, Universiti Putra Malaysia, Seri Kembangan, Selangor Malaysia; 6 Division of Applied Life Science, Gyeongsang National University, Jinju, South Korea

**Keywords:** Methanotrophs, *pmoA*, Methane, Agriculture, Oil palm, Tropical soil, Respectively

## Abstract

Oil palm (OP) plantations are gradually replacing tropical rainforest in Malaysia, one of the largest palm oil producers globally. Conversion of lands to OP plantations has been associated with compositional shifts of the microbial community, with consequences on the greenhouse gas (GHG) emissions. While the impact of the change in land use has recently been investigated for microorganisms involved in N_2_O emission, the response of the aerobic methanotrophs to OP agriculture remains to be determined. Here, we monitored the bacterial community composition, focusing on the aerobic methanotrophs, in OP agricultural soils since 2012, 2006, and 1993, as well as in a tropical rainforest, in 2019 and 2020. High-affinity methane uptake was confirmed, showing significantly lower rates in the OP plantations than in the tropical rainforest, but values increased with continuous OP agriculture. The bacterial, including the methanotrophic community composition, was modified with ongoing OP agriculture. The methanotrophic community composition was predominantly composed of unclassified methanotrophs, with the canonical (*Methylocystis*) and putative methanotrophs thought to catalyze high-affinity methane oxidation present at higher relative abundance in the oldest OP plantation. Results suggest that the methanotrophic community was relatively more stable within each site, exhibiting less temporal variations than the total bacterial community. Uncharacteristically, a 16S rRNA gene-based co-occurrence network analysis revealed a more complex and connected community in the OP agricultural soil, which may influence the resilience of the bacterial community to disturbances. Overall, we provide a first insight into the ecology and role of the aerobic methanotrophs as a methane sink in OP agricultural soils.

**Supplementary Information:**

The online version contains supplementary material available at 10.1007/s00248-021-01908-3.

## Introduction

Together, Malaysia and Indonesia are the major palm oil producers, contributing approximately 85% of the total palm oil production to meet the global food, pharmaceutical, and biofuel demands, and the production rate is anticipated to increase in the coming decades [1, 2]. Although breeding technology has improved palm oil yields (e.g., development of palms with higher oil content), this only served as an incentive to open up even more lands for the crop [2]. To this end, more than 16 million hectares of land primarily claimed from tropical rainforest, as well as peatlands and other croplands (e.g., old rubber plantations) have been planted to oil palm (OP) [1–3]. While the adverse impact of deforestation for palm oil production on animals and plants is well publicized (e.g., diversity loss; [4, 5]), the effects of the change in land use on the belowground microorganisms have received less attention. Despite being responsible for a multitude of soil processes, the response of microorganisms specifically those involved in the turnover of the potent greenhouse gases (GHGs; methane and nitrous oxide; [6]) to the land conversion and long-term continuous OP agriculture is less known. More recent work showed the effects of OP agriculture-induced changes to the microbial groups catalyzing nitrous oxide emission (nitrification, denitrification [7]), but knowledge on the communities responsible for methane turnover is still lacking.

Predominantly, well-aerated soils are a sink for atmospheric methane, but may also transiently emit methane when conditions turn anoxic (e.g., after a rainfall) [8–11]. In both instances, aerobic methanotrophs catalyze the oxidation of methane. While putative and specific canonical methanotrophs exhibit high-affinity methane oxidation, consuming methane at atmospheric concentrations, many canonical aerobic methanotrophs are thought to mediate low-affinity methane oxidation, consuming methane at higher concentrations; thus, the low-affinity methanotrophs typically reside at oxic-anoxic interfaces serving as a methane bio-filter [12–16]. The putative high-affinity methanotrophs have been clustered into alphaproteobacteria (e.g., upland soil cluster, USC-α) and gammaproteobacteria (e.g., USC-γ), based on their *pmoA* gene (encoding for the particulate form of the methane monooxygenase) [12, 17, 18]. Interestingly, studies recently showed that canonical “low-affinity” methane-oxidizers (e.g., *Methylosinus, Methylocystis, Methyloferulla, Methylocapsa*, *Methylosarcina*) can also oxidize and assimilate methane at (circum-)atmospheric concentrations [19–21]. However, it is likely that the canonical gammaproteobacterial methanotrophs (*Methylosarcina*) rely on periods of high methane availability to accumulate storage polymers, subsequently used as supplemental substrate in times of low (atmospheric) methane availability, as would be anticipated during alternate flooding and drying (fallow) regime during rice cultivation [19], but not in OP agriculture. Because of low methane availability restricting the population size, high-affinity methanotrophs relying on atmospheric methane alone are anticipated to be vulnerable to disturbances, in contrast to the low-affinity methanotrophs which are relatively more resilient to environmental perturbation (e.g., drought, N-fertilization[22, 23]). As such, OP agriculture may impose a significant effect on the methanotrophic community, potentially impairing their activity [24, 25]. While previous work indicated the presence of aerobic methanotrophs in an OP agricultural soil, documenting a steep methane gradient in the upper 10–15 cm of the soil [3], nearly nothing is known of the underlying methanotrophic diversity catalyzing methane oxidation and thus contributing to the regulation of the methane flux under OP agriculture.

Pairwise comparisons of pristine tropical rainforest and OP plantations have been performed to determine the impact of deforestation for OP agriculture, which showed a strong effect of the change in land use on the GHG fluxes and the total microbial community [6, 26, 27]. However, it remains to be determined how changes the microbial community with continuous OP agriculture and the consequences on microbially mediated soil processes. Here, we followed the development of the bacterial community, with focus on the methanotrophs, in OP agricultural soils since 2012, 2006, and 1993, as well as in a tropical rainforest soil in two consecutive years (January, 2019 and 2020). After confirming the methane uptake capacity of these soils, the response of the methanotrophic and total bacterial community composition to long-term OP agriculture were determined, based on the *pmo*A and 16S rRNA gene, respectively. Additionally, a 16S rRNA gene-based co-occurrence network analysis was performed to explore non-random interactions among the bacterial community. A more complex network with high connectivity is expected to form in relatively stable environments (i.e., tropical rainforest) in contrast to agriculture-impacted soils [28], but a study has since shown otherwise [26]. Hence, we refrain from making a priori postulations on the change in the network structure, comparing the tropical rainforest to the OP plantation soils.

## Methods

### Site Description, Soil Sampling, and Microcosm Incubation

The sampling sites, belonging to Universiti Putra Malaysia (UPM), are located in Seri Kembangan, Malaysia (2°59′00″N 101°43′17″E). These sites were formerly secondary forests which have been cropped to oil palm at different times since 2012 (7 years at the first sampling in 2019), 2006 (13 years), and 1993 (26 years), respectively, covering an area of 4 ha with 850 oil palms, 8 ha with 1079 oil palms, and 6 ha with 626 oil palms. Fertilization was performed in early August 2018 and late November 2019 with “Super K18,” containing an N:P:K:Ca:Mg ratio of 10:5:18:2.5:0.5 at an average rate of ~ 530 kg ha^−1^ (range, 410–650 kg ha^−1^). Because the oil palm plantations were reclaimed from designated UPM land within the same area over time (adjacent to one another), these sites experience similar local climatic conditions (i.e., equatorial climate without distinct seasonal fluctuations, and even humidity; [5]) and have similar mineral soil types (*Bungor* series soil profile) characterized by sandy loam soil texture in the upper 20 cm (Ap horizon) where the soil was sampled. Hence, these plantations are suitable to study the impact of long-term OP agriculture on the methanotrophs. Additionally, mineral soil (sandy loam) from a tropical rainforest in the Penang National Park, Malaysia (5°17′06″N 100°27′22″E), representing a pristine environment without agriculture impact was used for comparison. The tropical rainforest was a forest reserve before legally gazetted as a protected national park since 2003.

The soils were collected in mid-January 2019 and 2020. In the OP plantations, sampling was performed along a transect with increasing distance apart (start pointing, and approximately 1 m, 5 m, and 15 m) between the rows of oil palms; these samples were regarded as independent replicates per site (*n* = 4). Soils were collected approximately 0.5–1.0 m away from the base of the oil palms and from the upper 15 cm with a spade. The spade was rinsed with deionized water and 70% ethanol in between sampling. In the tropical rainforest, sampling was performed randomly from three sites located at least 15 m apart and was regarded as independent replicates. In all sites, the aboveground vegetation and leaf litter were discarded prior to sampling. After sampling, the soils were loosely sieved (4 mm) without applying pressure through the sieve to homogenize and remove large debris/roots, before being stored in ziplock bags and transported to the laboratory in a styrofoam box with ice. Upon arrival at the laboratory, an aliquot of the soil was immediately stored in the − 20 °C freezer till DNA extraction and soil physico-chemical characterization; the remaining soil was stored in the 4 °C fridge till microcosm incubation set up (within 36 h).

The capacity of the soils to consume methane, indicative of an active methanotrophic population, was initially verified after the first sampling in 2019. Thereafter, molecular analyses were applied to soils sampled in both years. The potential for soil methane uptake was performed in laboratory incubations. Each microcosm consisted of 6 g fresh weight soil in 260 ml opaque bottles (Sercon Limited, Cheshire, UK), sealed with a screw cap with a gas-tight septum. The headspace methane was adjusted to 20–30 ppm_v_ in air, and incubation was performed at 30 °C in the dark without shaking. Headspace methane was measured periodically over approximately two weeks to follow methane uptake. Upon methane depletion, the bottles were uncapped and left to aerate for 30 min before resuming incubation under ~ 1.3%_v/v_ headspace methane in air. Hence, methane uptake was determined under circum-atmospheric methane levels to detect high-affinity methane oxidation, as anticipated to occur in well-aerated soils [20, 29], and at higher methane levels to determine the potential for low-affinity methane oxidation.

### Determination of Headspace Methane and Selected Soil Physico-chemical Characteristics

Headspace methane was determined using gas chromatography (GC) coupled to a thermal conductivity and pulsed discharge helium ionization detector (7890B, Agilent Technologies, JAS GC systems, Moers, Germany). The rate of methane uptake was determined by linear regression from the slope of methane depletion. Total carbon and nitrogen concentrations were determined using an elemental analyzer (Vario EL, Elementar Analysen-Systeme, Hanau, Germany) from oven-dried (40 °C) and sieved (0.4 mm) soil. Total inorganic nitrogen (ammonium and nitrate) contents in the soil were determined in 2 M KCl (1:2 dilution) using standard colorimetric methods as described before [30]. Gravimetric water content (%) was determined after drying the soil in the 60 °C oven until constant weight. Soil pH and EC were determined using a pH meter (Mettler-Toledo, GmbH, Giessen, Germany) and an EC probe (Hanna Instruments, Langnau bei Reiden, Switzerland), respectively.

### DNA Extraction and pmoA-Based qPCR

DNA was extracted from the starting material using the PowerSoil DNA Isolation kit (Qiagen, Hilden, Germany) according to the manufacturer’s instructions and was used as template for the qPCR and amplicon sequencing. The *pmo*A gene-targeted qPCR was performed using the primer combination A189f/mb661r in duplicate per DNA extract, yielding six replicates per site and year. The qPCR assay was performed using a CFX Connect real-time PCR system (Biorad, München, Germany), with each reaction (total volume, 20 µl) consisting of 10 µl 2X SensiFAST SYBR mix (Bioline GmbH, Luckenwalde, Germany), 1.4 µl of forward and reverse primers each (5 pmol µl^−1^), 1 µl bovine serum albumin (5 mg ml^−1^; Sigma-Aldrich Chemie GmbH, Taufkirchen, Germany), 4.2 µl DNase- and RNase-free water (Thermo Fisher Scientific, Brunswick, Germany), and 2 µl template DNA. The PCR thermal profile consisted of an initial denaturation step at 95 °C for 3 min, followed by 45 cycles of denaturation at 95 °C for 10 s, annealing at 62 °C for 10 s, and elongation at 72 °C for 25 s. The melt curve was determined from 70 to 95 °C at 1 °C increment. The calibration curve (10^1^ to 10^8^ *pmoA* gene copies) was derived from a clone library [31]. Amplification specificity was determined from the melt curve and also verified in 1% agarose gel electrophoresis.

### pmoA and 16S rRNA Gene Amplicon Preparation for Illumina MiSeq Sequencing

The *pmoA* and 16S rRNA genes were respectively amplified using the A189f/mb661r and 341F/805R primer combinations for Illumina MiSeq sequencing. The contents of each *pmoA*- and 16S rRNA gene–targeted PCR reaction and PCR thermal profile were as detailed before [32]. After amplification, the *pmoA* and 16S rRNA gene PCR products were treated similarly. The amplicons were purified using the GeneRead Size Selection kit (Qiagen, Hilden, Germany), following verification on 1% agarose gel electrophoresis. A subsequent PCR was performed with 5 µl template from the first PCR to attach adapters to the gene amplicons, using Nextera XT Index Kit (Illumina, San Diego, USA); the reagents, reagent concentrations, and thermal profile for the second PCR are given elsewhere [32]. Next, the gene amplicons were purified using the MagSi-NGS^PREP^ Plus Magnetic beads (Steinbrenner Laborsysteme GmbH, Wiesenbach, Germany), and the purified amplicons were pooled at equimolar amounts (133 ng) for library preparation and Illumina MiSeq sequencing version 3 chemistry (paired-end, 600 cycles).

### pmoA Gene Sequencing Analysis

The same initial sequence analysis (i.e., assembly of the paired-end reads, sorting based on the length and quality of the primers [≤ 2 errors] and barcodes [≤ 1 error], and removal of chimera), performed in Mothur version 1.35.1 [33] to filter and obtain high-quality contigs, was applied to the *pmoA* gene sequences as detailed previously [32, 34]. After filtering, an average of 52,479 high-quality contigs per sample (total 1,364,461 contigs) was obtained from the initial 2,330,420 contigs generated by Illumina MiSeq sequencing. The high-quality *pmoA* gene sequences were classified using BLAST against the GenBank non-redundant (nr) database and the lowest common ancestor algorithm in MEGAN version 5.11.3, based on curated *pmoA* gene database and MEGAN tree, respectively, as described before [35]. The *pmoA* gene sequences could be affiliated to putative (without cultured representatives), and known methanotrophs at the family/genus level, whenever available. The relative abundance of the *pmoA* gene sequences was integrated along with the soil physico-chemical parameters in a redundancy analysis (RDA) to determine the variables significantly affecting the methanotrophic community composition and to visualize the (dis)similarity of the community between sites and over time (2019 and 2020 sampling). The *pmoA* gene sequence data matrix was initially analyzed using the detrended correspondence analysis (DCA) to evaluate the gradient size of the species (OTU level) distribution, revealing a linear distribution (length of gradient, < 3), which indicates that the best-fit mathematical model was the RDA. The RDA was constructed using the function “rda,” and the significant environmental factors were determined using the function “envfit” with 999 permutations using the Vegan package in the R statistics software environment [36]. The *pmoA* gene sequences were deposited at the National Center for Biotechnology Information (NCBI) under the project number PRJNA749621.

### 16S rRNA Gene Sequencing Analysis and Co-occurrence Network Analysis

The initial sequence processing, that is, merging of the paired-end reads using PEAR [37], followed by de-multiplexing, and quality control using the consensus method to remove chimera and low-quality sequences with DADA2 [38] was performed using QIIME 2 version 2019.10, as detailed before [32, 39]. After filtering and quality control, 1,442,928 high-quality contigs were obtained from the initial 2,072,668 (on average, 69,088 contigs per sample). Singletons and doubletons were removed, and samples were rarefied to 22,600 contigs, based on the sample with the lowest number of contigs. The contigs were classified at 97% similarity against the Silva database v.132 [40]. Compositional differences in the bacterial community between sites and over time and the influence of the environmental variables on the bacterial composition were determined by canonical correspondence analysis (CCA), based on the relative abundance of the OTUs. The OTUs are given to the finest classified taxonomic resolution (family/genus/species), whenever available. Like for the *pmoA* gene, the 16S rRNA gene sequence data matrix was initially analyzed using DCA, revealing a non-linear distribution (length of gradient > 3), which suggests that the best-fit mathematical model for the data was a CCA. The CCA was implemented using the function “cca,” and the significant environmental factors were determined using the function “envfit” with 999 permutations using the Vegan package in the R statistics software environment [36]. The 16S rRNA gene sequences were deposited at the National Center for Biotechnology Information (NCBI) under the project number PRJNA746287.

Additionally, a co-occurrence network analysis was performed to explore bacterial interaction under different land uses, based on the 16S rRNA gene diversity as reported before [26, 41, 42]. The network analysis also provides insight on the response and resilience of the interacting bacterial community to disturbances [32, 39]. Correlations between OTUs were calculated using the Python module “SparCC” [43]. Highly statistically significant (*p* < 0.01) and, hence, non-random true “SparCC” correlations with a magnitude of > 0.7 and < -0.7, respectively, indicating positive and negative correlations, were used for the network construction [44]. The *p*-values were derived from 99 permutations of random selections of the data tables. The network was constructed and the network topology was calculated with Gephi [45]. The networks were assessed based on their topological properties, that is, the number of nodes and edges, modularity, network diameter, average path length, degree, and clustering coefficient. These topological features have been benchmarked [42, 46], and the interpretation of the network topology is given in Table 1.

**Table 1:** Correlations and topological properties of microbial networks in the tropical rainforest and OP plantation soils sampled in 2019 and 2020

Network properties	2019				2020			
	Tropical rainforest	OP (since 2012)	OP (since 2006)	OP (since 1993)	Tropical rainforest	OP (since 2012)	OP (since 2006)	OP (since 1993)
Number of nodes^a^	701	1131	768	847	648	883	1004	909
Number of edges^b^	8166	18,229	7829	8558	9200	19,473	34,890	15,647
Positive edges^c^	6455 (79%)	12,261 (67%)	5458 (70%)	5721 (67%)	6887 (75%)	11,747 (60%)	21,947 (63%)	8991 (57%)
Negative edges^d^	1711 (21%)	5968 (33%)	2371 (30%)	2837 (33%)	2313 (25%)	7726 (40%)	12,943 (37%)	6656 (43%)
Modularity^e^	0.78	1.724	1.317	1.616	0.717	2.297	1.233	3.473
Network diameter^g^	20	10	15	14	19	12	14	12
Average path length^h^	6.302	4.545	5.172	6.007	5.115	3.586	3.850	4.126
Average degree^i^	23.29	32.23	20.38	20.20	28.39	44.10	69.50	34.42
Av. clustering coefficient^j^	0.365	0.304	0.446	0.465	0.388	0.437	0.457	0.385

^a^Microbial taxon (at genus level) with at least one significant (*P* < 0.01) and strong (SparCC > 0.7 or <  − 0.7) correlation

^b^Number of connections/correlations obtained by SparCC analysis

^c^SparCC positive correlation (> 0.7 with *P* < 0.01)

^d^SparCC negative correlation (< − 0.7 with *P* < 0.01)

^e^The capability of the nodes to form highly connected communities, that is, a structure with high density of between nodes connections (inferred by Gephi)

^f^A community is defined as a group of nodes densely connected internally (Gephi)

^g^The longest distance between nodes in the network, measured in number of edges (Gephi)

^h^Average network distance between all pair of nodes or the average length off all edges in the network (Gephi)

^i^The average number of connections per node in the network, that is, the node connectivity (Gephi)

^j^How nodes are embedded in their neighborhood and the degree to which they tend to cluster together (Gephi)

### Statistical Analyses

The level of significance (*p* < 0.05) of the measured variables (i.e., soil physico-chemical parameters, *pmoA* gene abundance, and methane uptake rates) between sites per year was determined using ANOVA in Sigmaplot version 12.5 (Systat Software Inc., USA) after testing for normal distribution (Shapiro–Wilk test).

## Results

### Methane Uptake Rates and the Abiotic Environment

**Fig. 1:**
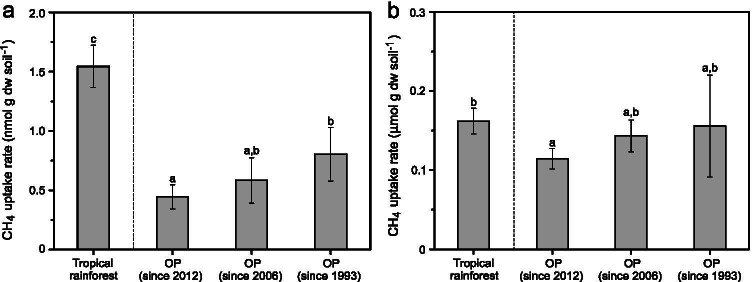
Potential high-affinity (**a**) and low-affinity (**b**) methane oxidation rates, as determined in incubations under low (< 30 ppm_v_) and high (~ 1.3%_v/v_) headspace methane concentrations, respectively (mean ± *sd*; *n* = 3–4). Letters indicate level of significance at *p* < 0.05. Note the different values of the *y*-axes

Soil methane uptake rate at circum-atmospheric concentration (< 30 ppm_v_), indicative of high-affinity methane oxidation [29], was significantly higher (*p* < 0.05) in the tropical rainforest relative to the OP plantations (Fig. 1). Within the OP plantations, the recently converted OP plantation (since 2012) showed a significantly lower (*p* < 0.05) soil methane uptake rate when compared to the oldest OP plantation (since 1993) (Fig. 1a). On the other hand, soil methane uptake rate at higher methane concentrations (~ 1.3%_v/v_), indicative of low-affinity methane oxidation, was largely comparable across all sites, except for the recent OP plantation (since 2012) exhibiting significantly (*p* < 0.05) lower values (Fig. 1b).

The soil pH was generally higher in the OP plantations than in the tropical rainforest, while gravimetric water content (GWC) was largely comparable across sites (Table 2). Total C and N were significantly higher in the tropical rainforest than in the OP plantations, with the exception of the oldest OP plantation sampled in 2020, whereby total C and N contents were comparable to the tropical rainforest (Table 2). The inorganic N (ammonium and nitrate) concentrations, however, showed different trends in soils sampled in 2019 and 2020. While inorganic N was on average (ammonium) or significantly higher (nitrate) in the tropical rainforest than in the OP plantation soils in 2019, the reverse was detected in 2020 (Table 2). Notwithstanding temporal variations, it is evident that OP agriculture induced changes to the soil abiotic environment and adversely affected the methanotrophic activity.

**Table 2:** Selected physico-chemical characteristics of the tropical rainforest and oil palm plantation soils

Site/sampling year	pH	GWC	EC	Total C	Total N	NH_4_^+^	NO_3_^−^
		(%)	(dS m^−1^)	(mg C g dw soil^−1^)	(mg N g dw soil^−1^)	(µg g dw soil^−1^)	(µg g dw soil^−1^)
Tropical rainforest							
2019	4.14 ± 0.17^a^	16.06 ± 5.26^a^	b.d.l	22.81 ± 8.12^b^	1.63 ± 0.40^b^	3.48 ± 0.67^a^	254.79 ± 32.74^c^
2020	4.62 ± 0.24^A^	15.04 ± 2.72^A^	b.d.l	29.39 ± 6.07^B^	2.29 ± 0.18^B^	1.04 ± 0.13^A^	90.58 ± 22.59^A^
OP (since 2012)							
2019	5.40 ± 0.14^b^	17.89 ± 1.64^a^	b.d.l	10.56 ± 1.58^a^	0.92 ± 0.17^a^	2.43 ± 0.69^a^	37.47 ± 14.65^a^
2020	5.36 ± 0.16^B^	16.26 ± 2.52^A^	b.d.l	14.09 ± 6.16^A^	1.01 ± 0.48^A^	6.24 ± 2.65^B^	398.38 ± 138.18^B^
OP (since 2006)							
2019	3.87 ± 0.05^c^	20.09 ± 1.61^a^	b.d.l	15.47 ± 2.83^b^	1.24 ± 0.22^b^	1.23 ± 0.64^a^	24.81 ± 4.39^b^
2020	5.23 ± 0.13^B^	21.27 ± 1.26^B^	b.d.l	19.20 ± 1.27^A^	1.40 ± 0.05^A^	4.23 ± 0.92^B^	574.14 ± 4.37^C^
OP (since 1993)							
2019	5.42 ± 0.20^b^	18.14 ± 2.16^a^	b.d.l	10. 76 ± 1.58^a^	0.92 ± 0.18^a^	2.12 ± 0.19^b^	59.86 ± 21.46^a^
2020	5.73 ± 0.21^C^	17.05 ± 2.21^A^	b.d.l	28.83 ± 1.99^B^	2.05 ± 0.09^B^	4.91 ± 0.49^B^	559.20 ± 36.54^B;C^

Results are given as mean ± *sd* (*n* = 3 for tropical rainforest soil; *n* = 4 for oil palm plantation soils). Level of significance at *p* < 0.05 (ANOVA) between sites per year (lower and upper case letters for 2019 and 2020, respectively)

*Abbreviations*: *GWC* gravimetric water content, *b.d.l.* below detection limit (EC, < 0.1 dS m^−1^)

### The pmoA-Based Methanotroph Abundance and Bacterial Community Composition

**Fig. 2:**
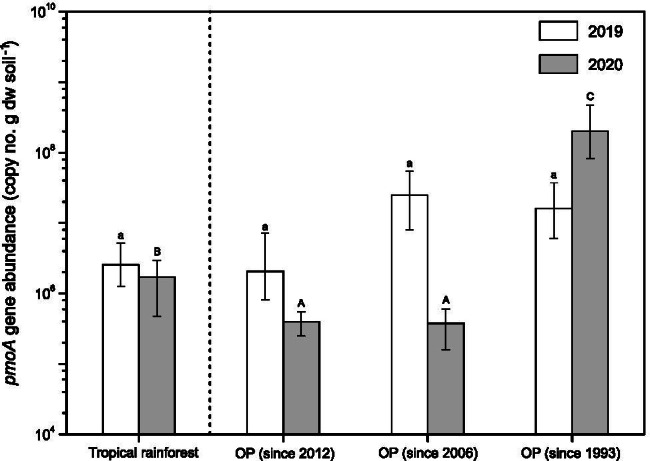
The *pmoA* gene abundance at the different sites (mean ± *sd*). The qPCR assay was performed in duplicate for each DNA extract, yielding a total of six replicates for the tropical rainforest soil, and eight replicates for the OP plantations soils. Lower and upper case letters indicate the level of significance (*p* < 0.05) between sites for 2019 and 2020, respectively. The lower detection limit of the qPCR assay was at approximately 10^5^ *pmoA* gene copy number per g dw soil

**Fig. 3:**
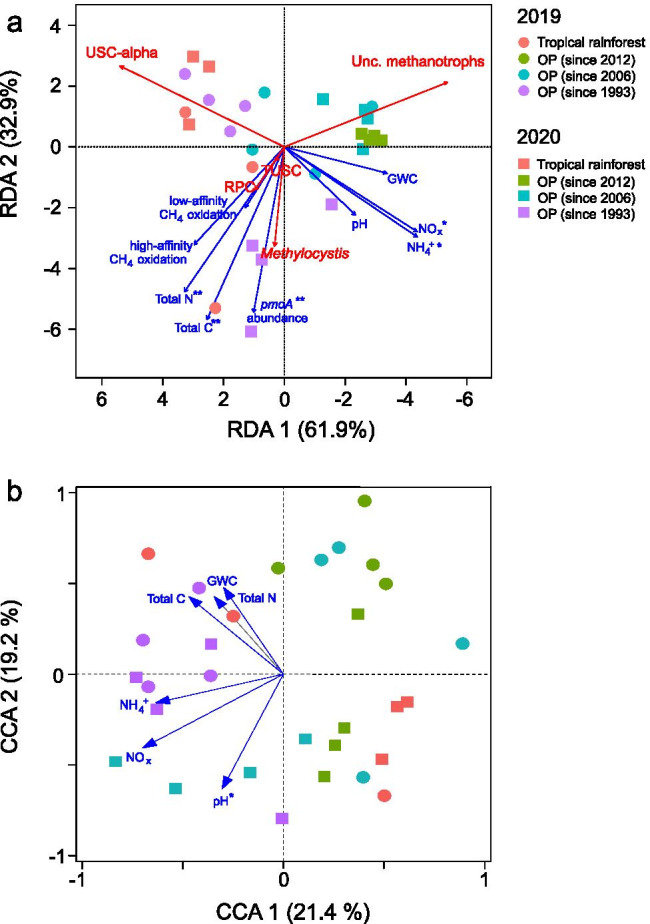
The composition of the methanotrophic (**a**) and total bacterial (**b**) community in the tropical rainforest and OP plantation soils and the influence of the environmental variables. A RDA was performed to visualize compositional (dis)similarity of the *pmoA* gene-based methanotrophic community between sites, while a CCA was performed for the 16S rRNA gene-based bacterial community, according to the best-fit mathematical model for the respective datasets. In **a**, the vectors in red represent the predominant methanotrophs. Also, note that *pmoA* gene sequencing was unsuccessful for the soil from the OP plantation (since 2012) in the 2019 sampling. In **a** and **b**, the level of significance is indicated by single asterisk (*p* < 0.05) and double asterisks (*p* < 0.01). The average relative abundance of the methanotrophic and bacterial community composition is given in the Supplementary Information (Figs. S1 and S2, respectively). Abbreviations: USC, upland soil cluster; TUSC, tropical upland soil cluster; RPC, rice paddy cluster; Unc., uncultured

The *pmoA* gene was enumerated to determine the methanotroph abundance. The *pmoA* gene abundance was on average (in 2019) or significantly (*p* < 0.05, in 2020) higher in the OP plantation since 1993, relative to the other sites (Fig. 2). Likewise, the *pmoA* gene was targeted, comparing the methanotrophic community composition in the tropical rainforest to the OP plantation soils (RDA; Fig. 3; Fig. S1). Proteobacterial methanotrophs affiliated to the tropical upland soil cluster (TUSC), rice paddy cluster (RPC), upland soil cluster-alpha (USC-α), *Methylocystis*, and as yet unclassified methanotrophs collectively represented the majority (77–90%) of the total community composition in all soils. Members of TUSC and RPC are putative high-affinity methanotrophs without cultured representatives, while members of USC-α are thought to be facultative high-affinity methanotrophs, closely affiliated to *Methylocapsa* [21, 47–49]. Some *Methylocystis* species harbor the pMMO2, enabling oxidation of methane at atmospheric levels [50, 51]. The methanotrophic community composition in the more recently converted OP plantations since 2012 and 2006 tended to cluster closely together and could generally be separated from the community in the oldest OP plantation and tropical rainforest, which were more dispersed, as revealed by the RDA (Fig. 3). Unclassified methanotrophs were correlated to the recently converted OP plantations, whereas the other predominant (putative) methanotrophs were favored in the tropical rainforest and oldest OP plantation soils. This suggests compositional shifts among the methanotrophs with ongoing OP agriculture.

The bacterial community composition was characterized based on the 16S rRNA gene diversity (Fig. 3). The predominant phyla include Proteobacteria, Acidobacteria, Actinobacteria, Chloroflexi, and Verrucomicrobia, together contributing > 78% to the total community (Fig. S2). The bacterial community composition was relatively more similar and consistent in the oldest OP plantation in both years, as indicated by the close clustering of the soils sampled in 2019 and 2020 (CCA; Fig. 3). On the other hand, the composition of the bacterial community in the other soils tended to cluster according to sampling time (2019 or 2020). This suggests that the bacterial community composition in these sites was relatively more prone to annual variations than the methanotrophic community composition.

### The 16S rRNA Gene-Based Co-occurrence Network Analysis

Potential bacterial interaction was explored by a co-occurrence network analysis, based on the 16S rRNA gene. Generally, the network analysis revealed a more complex and connected community under OP agriculture than in the tropical rainforest, as indicated by a higher number of nodes (i.e., at the OTU level), edges (i.e., connections), and to a lesser extent in the 2019 samples, a higher average degree (i.e., number of connections per node, or node connectivity) (Table 1; Fig. S3). The number of edges representing negative correlations was higher in the agricultural soils (Table 1). Accordingly, modularity (i.e., compartmentalization or the ability to form densely connected groups of nodes within the network) was consistently higher in the OP agricultural than tropical rainforest soils, whereas the reverse was detected for the average path length (i.e., average distance of the edges between nodes; Table 1). Within the agricultural soils, time after the introduction of OP had no apparent consistent effect on the network topology.

## Discussion

### The Impact of OP Agriculture on the Methanotrophic Activity and the Abiotic Environment

The soils showed capacity for high- and low-affinity methane uptake. However, methanotrophic activity was impaired under OP agriculture, as indicated by the significantly lower soil methane uptake particularly in the recently converted OP plantation since 2012 (Fig. 1). Although still adversely affected, increased soil methane uptake as OP plantation ages suggests a slow recovery in high-affinity methane oxidation, while low-affinity methane oxidation was already comparable to the rate exhibited in the tropical rainforest soil after 13 years of OP agriculture. Indeed, the methane sink function in well-aerated soils is sensitive to disturbances, including agricultural practices [52], requiring decades to recover to pre-agriculture levels [24], albeit climate-smart management strategies (e.g., usage of bio-based soil additives, cover crops) can abate agriculture-induced impact on soil methane uptake (e.g., [52, 53]. Comparably, low-affinity methane oxidation appears to be less vulnerable to agriculture-induced stressors and showed rapid recovery (e.g., N fertilization, salinization, desiccation-rewetting [23, 39, 54]).

To a large extent, trends in soil pH and total C and N were consistent over two consecutive years. Soil pH, a significant determinant of the bacterial community composition [55, 56] (Fig. 3), is often increased in OP plantations (e.g., through liming [57, 58]) to optimize conditions for crop growth (Table 2). The conversion to agricultural lands is also accompanied by reduced total C and N, through heightened decomposition of the soil organic matter during the change in land use [58–60]. Also, the belowground C stock is not continuously replenished with OP agriculture over time, in contrast to the accumulation of organic matter via litter input in the tropical rainforest soil [57, 61]. Nevertheless, oil palm fronds and processed fruit bunch are often distributed, albeit not homogenously, close to the base of the oil palms [62], as in our study sites, which may contribute to the soil organic matter in the long-term. The total C has been positively correlated to the methanotrophic activity and/or community composition [63, 64]. With the exception of a few facultative methanotrophs (e.g., USC-α, *Methylocystis*, *Methylocapsa* [47, 65]), the majority of methanotrophs assimilate methane for growth. Hence, increased C likely imposed an indirect effect, fueling methanogenesis in anoxic microniches, and, in turn, provides the methanotrophs with their main substrate. On the other hand, year-to-year variation in the inorganic N (ammonium and nitrate) concentrations is likely attributable to the interval between fertilization and soil sampling (Table 2). The soils were sampled in mid-January in 2019 and 2020, but fertilization was performed in early August (2018) and late November (2019). Therefore, the shorter interval prior to sample collection after fertilization in 2020 (< 2 months) when compared to 2019 (~ 5.5 months) may partly explain the higher ammonium and, correspondingly, nitrate concentrations (via nitrification) in the OP plantation soils. Consistent with previous studies (see review [6]), changes in the soil physico-chemical parameters are inherent to OP agriculture, exerting an effect on microbially mediated soil processes, including the methanotrophic activity.

### The Impact of Oil Palm Agriculture on the Methanotrophic Abundance and Bacterial Community Composition

The *pmoA* gene abundance was 1–2 orders of magnitude higher in the oldest OP plantation, when compared to the tropical rainforest and more recently converted OP plantations, indicating the long-term recovery of the methanotroph abundance with ongoing OP agriculture spanning almost three decades. Assuming that a methanotroph harbors two *pmoA* gene copies [66], the apparent cell-specific activity was determined considering the methanotroph abundance and high-affinity methane uptake rate, given that these are predominantly well-aerated soils serving as a sink for atmospheric methane. The mean apparent cell-specific activity was highest in the tropical rainforest (5.03 × 10^−17^ mol CH_4_ h^−1^ cell^−1^), whereas values ranged from 1.89 × 10^−17^ to 1.94 × 10^−18^ mol CH_4_ h^−1^ cell^−1^ in the OP plantations soils (Table S1). These values are lower than would be expected to be sufficient for growth on atmospheric methane alone (10^−16^ mol CH_4_ h^−1^ cell^−1^ range [20, 67]). Hence, methane produced at anoxic microsites and/or alternative substrates (e.g., acetate) may have also supported methanotrophic growth. This is corroborated by the *pmoA* gene sequencing analysis, revealing the presence of potential facultative methanotrophs in these soils (USC-α, *Methylocystis*; Fig. 3). Hence, although methanotroph abundance increased, the methanotrophic activity per cell decreased with long-term OP agriculture.

The bacterial community, including the methanotrophs, showed compositional shifts with ongoing OP agriculture. However, the methanotrophic community composition, more pronounced in the recent OP plantations (since 2012 and 2006), tended to cluster according to site, while the total bacterial community in all sites (exception, foremost OP plantation), clustered according to sampling year (2019 and 2020; Fig. 3). This suggests that the methanotrophic community was relatively more stable within each site and was less affected by year-to-year variations than the overall community, during early (< 13 years) OP agriculture. Although the sampling sites are characterized by even climatic conditions, this trend can be further substantiated, documenting high-resolution short-term temporal dynamics of the bacterial community composition in future studies. Accordingly, the bacterial community composition, including the methanotrophs, can be profoundly influenced by the soil physico-chemical parameters and land use [25, 68–71], also shown here (Fig. 3), with land use exerting a stronger impact specifically on the methanotrophs [72]. More generally, the compositional differences detected comparing the tropical rainforest and OP plantation soils may reflect on the anticipated changes of the bacterial community following deforestation for oil palm agriculture.

### The Emergence of a Complex Interaction Network Under OP Agriculture

The co-occurring network analysis revealed a generally more complex and connected interaction network under OP agriculture than in the tropical rainforest, which may be partly attributable to increased competition for more limited resources in the agricultural soils, fostering interaction among community members [73]. Indeed, total C and N were on average or significantly lower in the OP plantation than in the tropical rainforest soils (Table 2). Also, increased pH following OP agriculture is thought to promote microbial proliferation, likely increasing their co-occurrence [6]. The agricultural soils also exhibited a more modular interaction network, forming more compartments within the network; a highly compartmentalized network is anticipated to localize stressor-induced effects in times of disturbances [74]. The relevance of a modular network structure in mitigating the effects of stress on community functioning was corroborated in a recent study, where a reduction in network modularity was concomitant to significantly impaired methane uptake rates, following ammonium-induced stress [32]. Accordingly, the average path length was lower in the OP agricultural soil than in the tropical rainforest (Table 1). Having an interaction network with a shorter average path length implies a tighter network, enabling the community to respond rapidly to stressors [75, 76]. Overall, the topological features indicate a more complex and connected community and also predict the network to respond more efficiently to disturbances under OP agriculture than in the tropical rainforest. This may seem counterintuitive, given that a complex network with high connectivity is characteristic of a stable environment, relatively free from anthropogenic influence (e.g., pristine peatlands, tropical rainforest, and long-term restored former agricultural lands [28, 44, 77, 78]). However, results are consistent with a previous work comparing co-occurrence networks in a pristine tropical rainforest to logged rainforest and oil palm agricultural soil [26]. Hence, the causative mechanism driving microbial interaction in OP agriculture remains unclear. Nevertheless, OP agriculture likely exerted a pervasive effect, leaving an imprint on the ecological interaction of the soil bacterial community.

## Conclusions

Here, we showed that the methanotrophic communities in the tropical rainforest and OP agricultural soils were predominantly represented by the putative high-affinity methane-oxidizers and as yet uncultivated methanotrophs, indicating a wealth of under-explored methanotrophic diversity in these environments. Our findings showed that ongoing OP agriculture modified the composition of the methanotrophs, as well as the capacity for high-affinity methane oxidation. Admittedly, our study may not have captured variations in the (a)biotic parameters at high temporal resolution in the short-term, which will be addressed in future field studies. Nevertheless, we determined the long-term effects of OP agriculture spanning over almost three decades and provide a first insight on the methanotrophic community and potential for methane oxidation in OP agricultural soils. Besides, we reinforced previous findings showing the uncharacteristic trend of increased network complexity and connectivity under OP agriculture. Understanding the ecology of the methanotrophs and their role as a methane sink is crucial to devise climate-smart strategies to mitigate GHG emissions in OP plantations.

## Supplementary Information

Below is the link to the electronic supplementary material.

Supplementary file1 (PDF 4384 KB)

## Data Availability

The nucleotide sequence data reported are available in the NCBI databases under the project numbers PRJNA746287 and PRJNA749621 for the 16S rRNA and *pmoA* gene, respectively.

## Code Availability

Not applicable.

All authors have seen and approved the final version submitted.
