# Supplementary material for: Aerobic Methanotrophy and Co-occurrence Networks of a Tropical Rainforest and Oil Palm Plantations in Malaysia
Source: Microb Ecol. 2021 Oct 30;84(4):1154–65. doi: 10.1007/s00248-021-01908-3 (PMC9747831; doi:10.1007/s00248-021-01908-3)
Supplement: Supplementary file 1 — Supplementary file1 (PDF 4384 KB) [file 248_2021_1908_MOESM1_ESM.pdf]

Supplementary Information

Aerobic methanotrophy and co-occurrence networks of a tropical rainforest and oil palm plantations in Malaysia.

Adrian Ho[1*], Ali Tan Kee Zuan[2], Lucas W. Mendes[3], Hyo Jung Lee[4], Zufarzaana Zulkeflee[5], Hester van Dijk[1], Pil Joo Kim[6], Marcus A. Horn[1].

[1]Leibniz Universität Hannover, Institute for Microbiology, Hannover, Germany.

[2]Universiti Putra Malaysia, Department of Land Management, Faculty of Agriculture, Selangor, Malaysia.

[3]University of São Paulo CENA-USP, Center for Nuclear Energy in Agriculture, São Paulo, Brazil.

[4]Kunsan National University, Department of Biology, Gunsan, South Korea.

[5]Universiti Putra Malaysia, Department of Environment, Faculty of Forestry and Environment, Selangor, Malaysia.

[6]Gyeongsang National University, Division of Applied Life Science, Jinju, South Korea.

*For correspondence: Adrian Ho (adrian.ho@ifmb.uni-hannover.de).

Included:

- **Supplementary table.**
- **Supplementary figures and figure captions.**

Supplementary Table S1: Calculated mean apparent cell-specific methane uptake rate in the tropical rainforest and OP plantation soils sampled in 2019.

Land-use	Apparent cell-specific activity ($mol\ CH_4\ h^{-1}\ cell^{-1}$)
Tropical rainforest	5.03×10^{-17}
OP (since 2012)	1.80×10^{-17}
OP (since 2006)	1.94×10^{-18}
OP (since 1993)	1.10×10^{-18}

Supplementary figure legends

Figure S1 The methanotrophic community composition, based on the *pmoA* gene diversity (mean, n=3 or 4). OTUs with < 0.5 % are grouped as 'Others'. Abbreviations: USC, upland soil cluster; TUSC, tropical upland soil cluster; RPC, rice paddy cluster; Unc., uncultured

Figure S2 The bacterial community composition at the phyla level, based on the 16S rRNA gene diversity (mean, n=3 or 4)

Figure S3 Co-occurrence network analysis in the tropical rainforest (a), and oil palm plantation soils since 2012 (b), 2006 (c), and 1993 (d) sampled in 2019 and 2020. Network topology are given in Table 1. Only significantly correlated nodes ($p<0.01$) with a `SparCC` correlation of a magnitude of >0.7 (positive correlations) or <-0.7 (negative correlations) were used to construct the networks. Each node represents a taxon at the OTU-level, and the size of the node corresponds to the number of connections. Photos are curtesy of Adrian Ho and Ali Tan Kee Zuan.

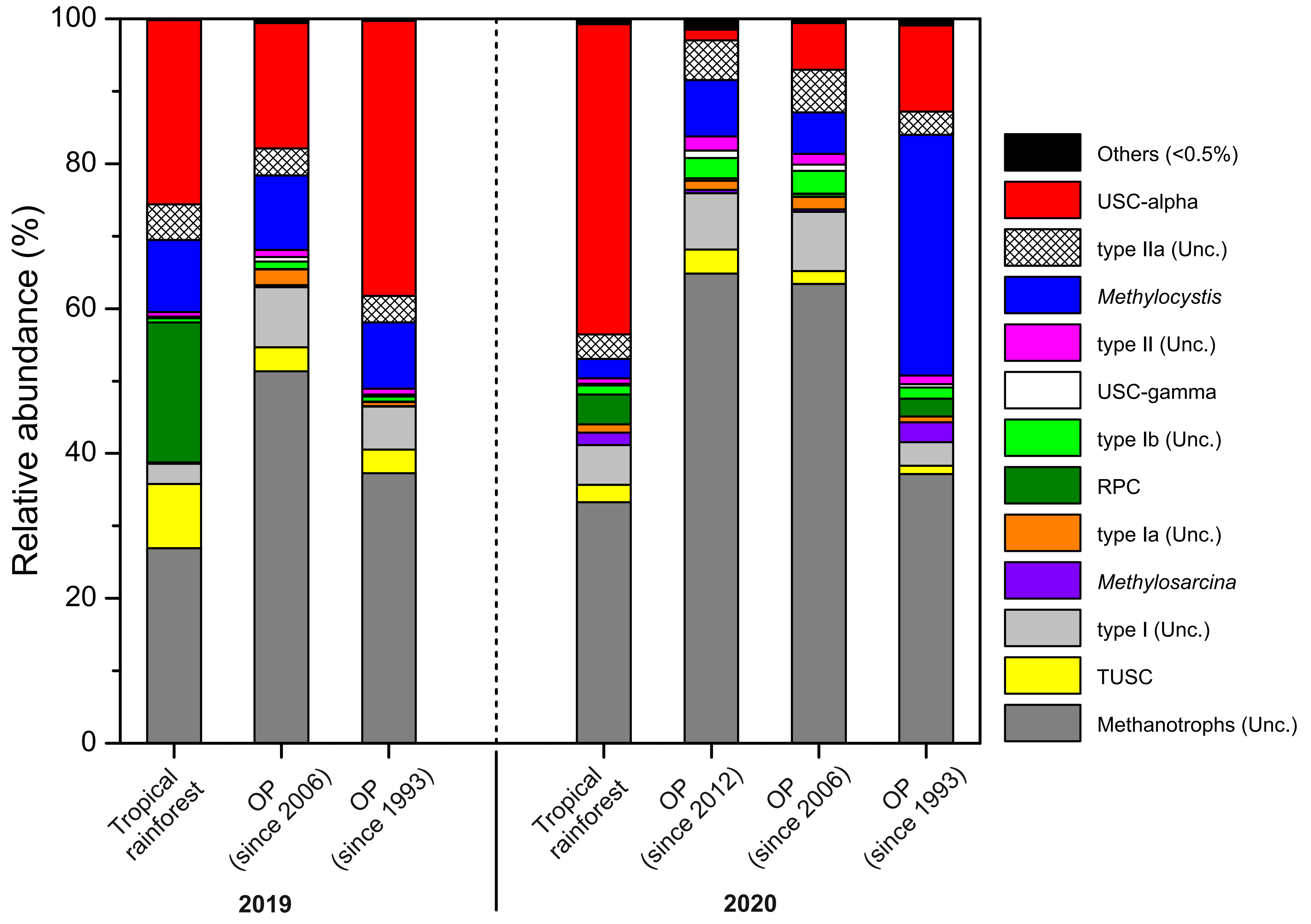

Relative abundance (%)
100
80
60
40
20
0
Tropical rainforest
OP (since 2006)
OP (since 1993)
Tropical rainforest
OP (since 2012)
OP (since 2006)
OP (since 1993)
2019
2020
Others (<0.5%)
USC-alpha
type IIa (Unc.)
Methylocystis
type II (Unc.)
USC-gamma
type Ib (Unc.)
RPC
type Ia (Unc.)
Methylosarcina
type I (Unc.)
TUSC
Methanotrophs (Unc.)

Figure S1

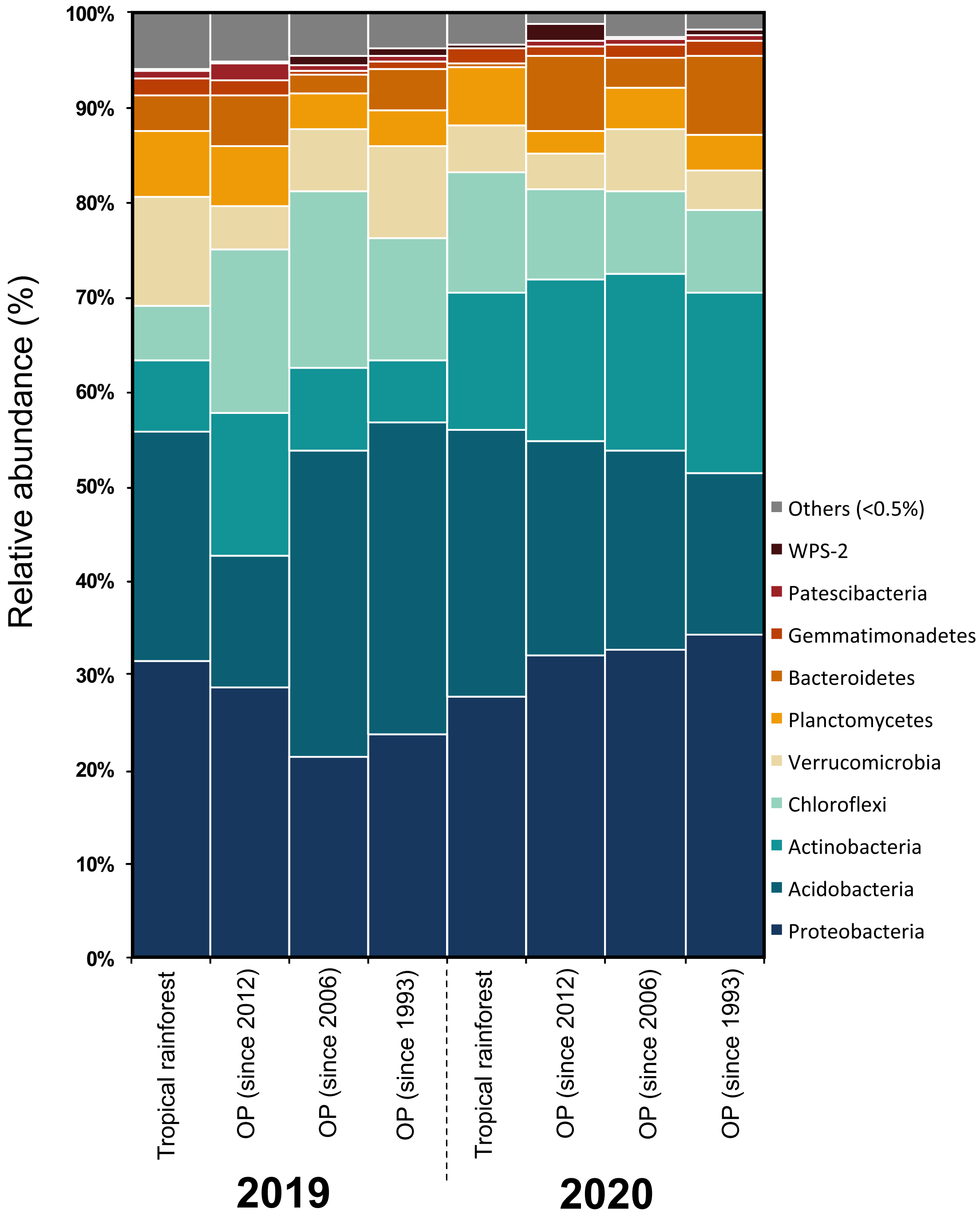

Relative abundance (%)
100%
90%
80%
70%
60%
50%
40%
30%
20%
10%
0%
Others (<0.5%)
WPS-2
Patescibacteria
Gemmatimonadetes
Bacteroidetes
Planctomycetes
Verrucomicrobia
Chloroflexi
Actinobacteria
Acidobacteria
Proteobacteria
Tropical rainforest
OP (since 2012)
OP (since 2006)
OP (since 1993)
Tropical rainforest
OP (since 2012)
OP (since 2006)
OP (since 1993)
2019
2020

Figure S2

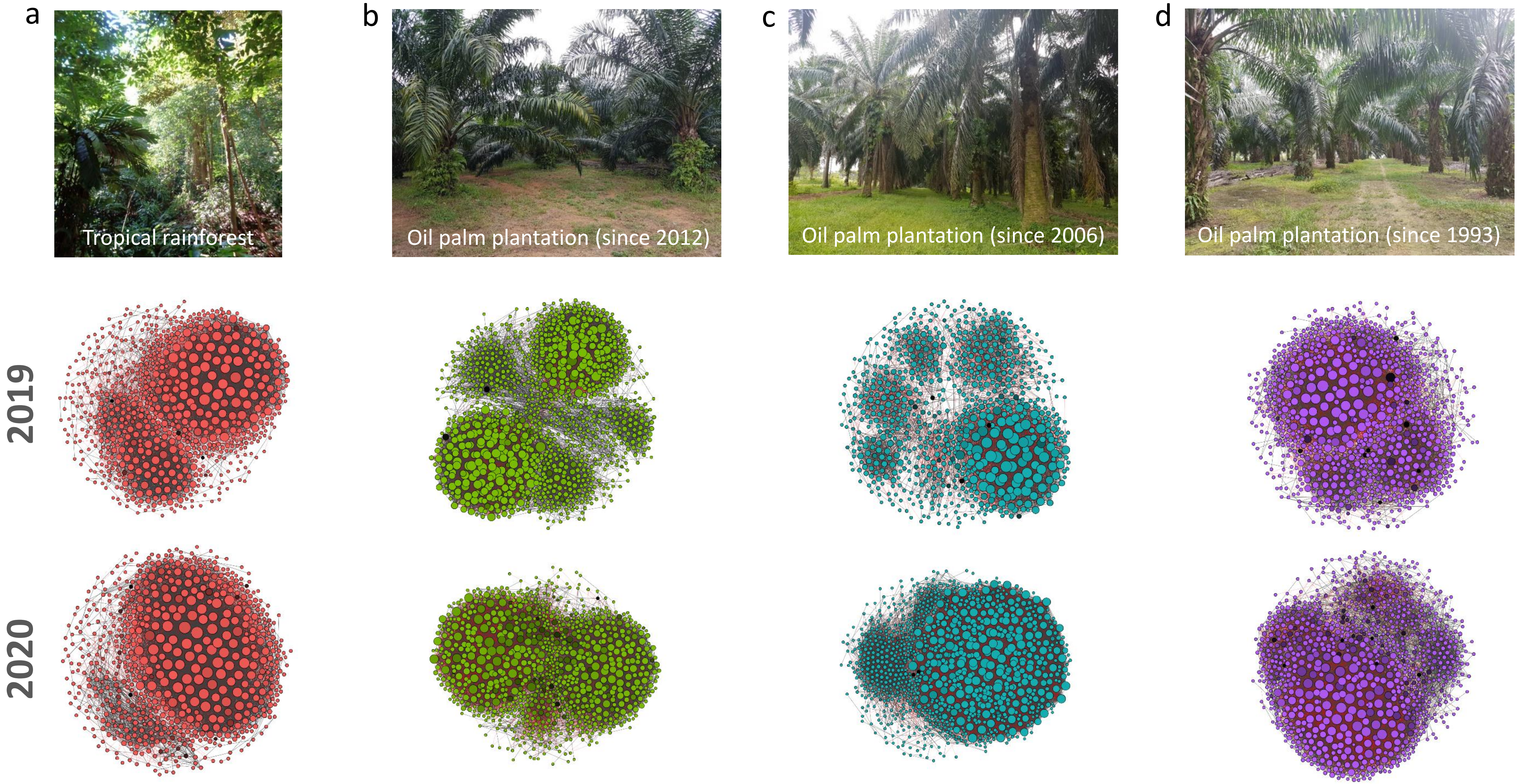
a
Tropical rainforest
b
Oil palm plantation (since 2012)
c
Oil palm plantation (since 2006)
d
Oil palm plantation (since 1993)
2019
2020

Figure S3
